# Non-Traumatic Intramuscular Hip Hematoma in a Cirrhotic Patient With Hepatocellular Carcinoma and Portal Vein Thrombosis Treated With Sorafenib and Low Molecular Weight Heparin

**DOI:** 10.7759/cureus.14818

**Published:** 2021-05-03

**Authors:** Christos Sotiropoulos, Konstantinos Thomopoulos

**Affiliations:** 1 Gastroenterology, University General Hospital of Patra, Patra, GRC

**Keywords:** hip hematoma, cirrhosis, hepatocellular carcinoma, portal vein thrombosis, sorafenib, low molecular weight heparin

## Abstract

Hepatocellular carcinoma (HCC) is a common neoplasm amongst cirrhotic patients and portal vein thrombosis (PVT) is an often found complication. Sorafenib and low molecular weight heparin (LMWH) are considered part of the gold-standard treatment of such patients. Spontaneous intramuscular hematomas of the limbs induced by these agents, as an adverse event, are generally rare. We present a 66-year-old male patient with liver cirrhosis, HCC and PVT treated with Sorafenib and LMWH who developed a non-traumatic hip hematoma. Simple elevation of the lower limb and blood-products infusion was successful in leading to resolution of the symptoms. As the popularity of these agents increases, healthcare providers need to be aware of such treatment adverse events.

## Introduction

Hepatocellular carcinoma is the fourth most frequent neoplasm and the second most common cause of cancer death in the world [1]. The majority of the patients with HCC have liver cirrhosis, usually due to hepatitis B or hepatitis C virus infection or chronic alcohol consumption [1]. portal vein thrombosis (PVT) is a common complication of HCC with a prevalence from 10% to 60% [2]. It represents the most common form of macrovascular invasion of the carcinoma (neoplastic thrombus) or develops in the context of a hypercoagulable state [2].

Sorafenib, a small molecule with specific activity against vascular endothelial growth factor receptor, is used in PVT from neoplastic thrombus [2] and is considered a gold-standard treatment for advanced HCC. Sorafenib’s most common toxicities include hand-foot skin reaction (palmar-plantar erythrodysaesthesia) and rash, diarrhea, fatigue and weight loss, abnormal liver function and hypertension. Infrequent bleeding events have been reported, especially in some patients taking anticoagulation therapy while on Sorafenib therapy [3]. On the other hand, in non-malignant PVT, anticoagulation remains the mainstay of therapy with low molecular weight heparin (LMWHs) being the most frequent used agents [4]. Bleeding is the primary complication of anticoagulant therapy and includes major or life-threatening hemorrhage and minor bleeding events [5]. The most common bleeding events include intracranial hemorrhage and intracerebral bleeding, gastrointestinal bleeding, intraocular bleeding and ecchymosis or bruises [5].

In this case report, we present a 66-year-old male cirrhotic patient with HCC and PVT who developed a spontaneous non-traumatic hematoma on his left hip while treated with Sorafenib and LMWH. This case is of great interest regarding the unusual location of the hematoma and the non-traumatic etiology. To the best of our knowledge, this is the first report of a Sorafenib/LMWH induced non-traumatic hip hematoma in a cirrhotic patient.

## Case presentation

We report a 66-year-old male patient with liver cirrhosis, multifocal HCC and PVT in the setting of chronic ethylation treated with Sorafenib and LMWH. The patient was admitted to the hospital’s emergency department due to non-traumatic left hip pain. The patient on the examination was hemodynamically stable and fever-free. The findings from the physical examination included moderate ascites, no signs of encephalopathy, a swelling (non-fermented edema) and warm thigh, without skin lesions or deformity and with no signs of critical limb ischemia.

The laboratory values revealed hypochromic-microcytic anemia (Hct: 18.80%, Hb: 6.20 g/dl, MCV: 67.60 fl, MCH: 22.30 pg), impaired coagulation mechanism (INR: 2.09, aPTT: 52.5 sec), acute kidney injury (Cre: 2.5 mg/dl, Urea: 81 mg/dl), abnormal liver function tests (TBL: 10.11 mg/dl, DBL: 9.29 mg/dl, AST: 395 U/l, ALT: 102 U/l, γ-GT: 220 U/l, ALP: 183 U/l, ALB: 3.0 gr/dl) and a Child-Pugh Class C patient.

**Figure 1:**
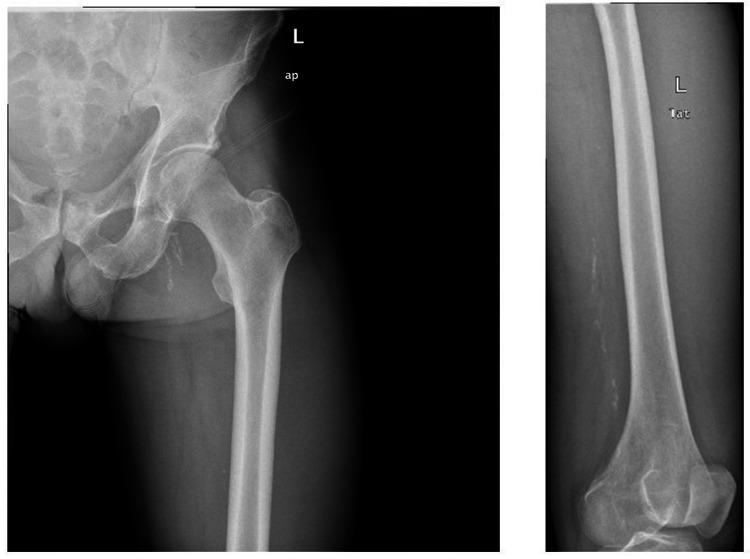
Hip and pelvic X-ray examination with no bone lesions.

The hip and pelvic X-ray examination revealed no bone lesions (Figure 1).

**Figure 2:**
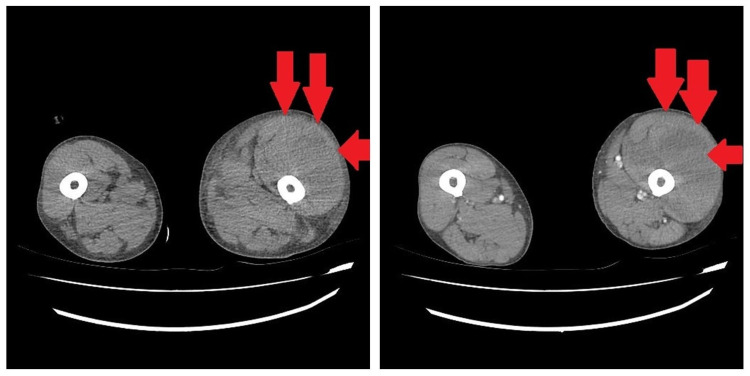
Extensive hematoma (red arrows) on the anterior-lateral surface of the left thigh (without an image of active extravasation) revealed on a lower extremity CT angiography.

Due to the clinical laboratory findings and the history of anticoagulant treatment left lower extremity hematoma was suspected, so the patient underwent a lower extremity CT angiography which showed extensive hematoma (red arrows) on the anterior-lateral surface of the left thigh without an image of active extravasation (Figure 2).

The patient was treated conservatively with cessation of anticoagulant and Sorafenib therapy and with transfusion of blood derivatives and an orthopedic evaluation was performed where left lower extremity elastic bandage was placed. The patient had an uncomplicated hospitalization with gradual absorption of the hematoma, without signs and symptoms of compartment syndrome.

## Discussion

HCC and PVT are well-defined and studied medical entities. Management guidelines and therapeutic options are well-established and the appearance of treatment-related adverse events is a common subject of the daily medical practice. Although there are common side effects, there are also rare adverse events, as described in this case report.

Sorafenib is an antineoplastic agent that is known to modestly prolong the median overall survival of patients with HCC and its side effects have been thoroughly studied [2]. The most common adverse events include skin reactions and rashes, weight loss, diarrhea, fatigue, abnormal liver function and hypertension. Bleeding events have been infrequently reported, mostly in some patients taking anticoagulation therapy while on Sorafenib therapy [3]. On the other hand, LMWH therapy has been widely utilized for many years and is considered to be one of the leading causes of medication-related side effects [6]. Bleeding is the primary complication of anticoagulant therapy and includes major and minor bleeding events [5]. The most common bleeding events are intracranial hemorrhage, gastrointestinal bleeding and ecchymosis or bruises [5]. Thus, healthcare providers and patients considering this therapy must adopt standard therapeutic strategies to achieve prevention and treatment of such adverse events [6].

Trauma is the most common cause that can lead to muscle hematomas, especially while on anticoagulation use [7]. Non-traumatic intramuscular hematomas induced by LMWH or Sorafenib are rare and to our knowledge, this is the first reported case of a hip hematoma under such circumstances. We utilised non-operative measures for our patient as there was no clinical and radiological evidence of continuing bleeding, nerve compression or compartment syndrome. Finally, elastic bandaging and simple elevation of the limb were successful measures to resolve the symptoms and the patient was discharged from the hospital after a few days of hospitalization.

Little is known about the management of LMWH/Sorafenib-induced spontaneous hematomas, although consensus shows that such events can usually be faced non-operatively with cessation of the anticoagulation treatment, intravenous fluid infusion and blood-products administration [7]. Upon review of the published literature, there is no similar case to ours.

## Conclusions

Concluding, Sorafenib and anticoagulants are frequently used medical therapies for cirrhotic patients, so strict policies and evidence-based strategies for the management of bleeding events need to be established. Rapid assessment of the cause, the location and the severity of the bleeding and appropriate measures to control the hemorrhage are crucial. Several cases of bleeding events are present in the current literature, but the significance of our case is that demonstrates an extremely rare case of a localized hematoma under unusual circumstances. This case highlights the importance of awareness of the atypical presentation that medication-induced side effects may have.
